# The Protective Effect of Resveratrol on Concanavalin-A-Induced Acute Hepatic Injury in Mice

**DOI:** 10.1155/2015/506390

**Published:** 2015-05-24

**Authors:** Yingqun Zhou, Kan Chen, Lei He, Yujing Xia, Weiqi Dai, Fan Wang, Jingjing Li, Sainan Li, Tong Liu, Yuanyuan Zheng, Jianrong Wang, Wenxia Lu, Qin Yin, Yuqing Zhou, Jie Lu, Hongfei Teng, Chuanyong Guo

**Affiliations:** ^1^Department of Gastroenterology, Shanghai Tenth People's Hospital, Tongji University School of Medicine, Shanghai 200072, China; ^2^The First Clinical Medical College of Nanjing Medical University, Nanjing 210029, China; ^3^The First Affiliated Hospital of Soochow University, Suzhou 215006, China; ^4^Department of General Surgery, Shanghai Tenth People's Hospital, Tongji University School of Medicine, Shanghai 200072, China

## Abstract

*Pharmacologic Relevance.* Resveratrol, an antioxidant derived from grapes, has been reported to modulate the inflammatory process. In this study, we investigated the effects of resveratrol and its mechanism of protection on concanavalin-A- (ConA-) induced liver injury in mice. *Materials and Methods.* Acute autoimmune hepatitis was induced by ConA (20 mg/kg) in Balb/C mice; mice were treated with resveratrol (10, 20, and 30 mg/kg) daily by oral gavage for fourteen days prior to a single intravenous injection of ConA. Eight hours after injection, histologic grading, proinflammatory cytokine levels, and hedgehog pathway activity were determined. *Results.* After ConA injection, the cytokines IL-2, IL-6, and TNF-*α* were increased, and Sonic hedgehog (Shh), Glioblastoma- (Gli-) 1, and Patched (Ptc) levels significantly increased. Pretreatment with resveratrol ameliorated the pathologic effects of ConA-induced autoimmune hepatitis and significantly inhibited IL-2, IL-6, TNF-*α*, Shh, Gli-1, and Ptc. The effects of resveratrol on the hedgehog pathway were studied by western blotting and immunohistochemistry. Resveratrol decreased Shh expression, possibly by inhibiting Shh expression in order to reduce Gli-1 and Ptc expression. *Conclusion.* Resveratrol protects against ConA-induced autoimmune hepatitis by decreasing cytokines expression in mice. The decreases seen in Gli-1 and Ptc may correlate with the amelioration of hedgehog pathway activity.

## 1. Introduction

Liver disease remains a significant global health problem. Liver impairment is multifactorial and may result from viral infection, chronic excess alcohol use, hepatotoxins such as ibuprofen, an atherogenic high fat diet, ischemia-reperfusion injury, transplant, and surgical models, as well as radiation [1]. Indeed, liver disease remains a significant cause of morbidity and mortality globally [2–4]. Thus, there is an urgent need to discover hepatoprotective agents. A number of compounds of botanical origin have been investigated for their ameliorative effect in liver injury disease, in both experimental and clinical situations. For example, flavonoids and other natural products have been described to possess beneficial hepatic effects [5–7].

Resveratrol (3,4′,5-trihydroxystilbene) is present in more than 70 plant species, including grapes, peanuts, berries, and red wine and possesses beneficial antioxidant, anti-inflammatory, antimutagenic, and anticarcinogenic properties [8, 9]. The phytochemical resveratrol has shown considerable promise as a potential hepatoprotective agent [10]. Several studies have investigated the use of resveratrol in chronic illnesses such as arthritis, diabetes, neoplastic, and neurodegenerative diseases [11–13]. Resveratrol provides protection against ongoing liver damage resulting from hepatotoxins such as acetaminophen, ethanol, and carbon tetrachloride (CCl_4_) [14–16]. A recent study demonstrated that 2 weeks of resveratrol administration in rats prevented CCl_4_-induced lipid peroxidation, as evidenced by decreased MDA content. Thus, the antioxidant and anti-inflammatory properties of resveratrol play a prominent hepatoprotective role [17].

The hedgehog (Hh) signaling pathway plays a crucial role in vertebrate embryogenesis, by controlling cell fate, proliferation, survival, and differentiation. The level of Hh pathway activation appears to be proportional to the severity and duration of liver injury in both rodents and humans [18]. Mature hepatocytes themselves do not express all of the Hh proteins and are not capable of responding to Hh ligands [19]. However, many neighboring cells are Hh-responsive, including resident hepatic cell populations that are most engaged in liver remodeling (e.g., liver myofibroblasts, hepatic progenitors, hepatic satellite cells, immature cholangiocytes, endothelial cells, and T lymphocytes) [18–26]. The proximity of various types of Hh producing and Hh responding cell types in damaged livers suggests that Hh pathway activation coordinates complex autocrine and paracrine signaling loops that help to orchestrate remodeling and reconstruction of the injured liver. Jung et al. reported Hh signaling to be activated in cells that participate in the ductular reaction that is elicited by chronic alcohol-induced liver injury in mice and humans. The cumulative data indicate that Hh pathway activation is a common feature of various types of liver injury that mobilize hepatic progenitor populations [20].

Accordingly, this study was undertaken to examine the potential protective effects of resveratrol against liver damage caused by concanavalin-A (ConA) in mice using different biochemical parameters and histopathologic studies. ConA-induced liver injury is a mouse model of immune-mediated liver injury that resembles viral and autoimmune hepatitis in humans [12]. Intravenous injection of ConA into mice activates T cells which infiltrates the liver, leading to the subsequent process of hepatocyte apoptosis and necrosis, finally increases the level of plasma alanine aminotransferase (ALT) [27–29]. The activation of T cells by ConA results in increased levels of inflammatory cytokines, including tumor necrosis factor- (TNF-) *α*, interferon- (IFN-) *γ*, and interleukin- (IL-) 6 [13, 28, 29].

Clearly, there is a critical need to explore novel and alternative approaches for the treatment of liver disease. The structural determinants of the properties of resveratrol are obscure and its effects have not been tested in the model of acute liver damage induced by ConA. We aimed to investigate whether resveratrol was capable of preventing ConA-induced liver injury, as well as its molecular mechanism. We report that resveratrol can show functional and morphologic improvement by decreasing Gli-1 and Ptc expression, as well as inflammatory mediator expression in mice with ConA-induced liver injury.

## 2. Materials and Methods

### 2.1. Reagents and Drug Administration

ConA and resveratrol were purchased from Sigma-Aldrich (St. Louis, MO, USA) and stored at 4°C. Resveratrol was suspended in 0.5% carboxymethylcellulose solution and prepared separately at 1, 2, and 3 g/mL concentrations. Resveratrol was thoroughly mixed before the mice were fed; each mouse was fed 0.1 mL/10 g weight, in the following concentrations: 10, 20, and 30 mg/kg. The antibodies used for immunoblotting and immunohistochemical staining were anti-TNF-*α*, anti-IL-2, IL-6, CD4, F4/80, Shh, Gli-1, and Ptc (Santa Cruz Biotechnology, Dallas, TX, USA). ConA was dissolved in pyrogen-free physiological saline and intravenously injected at a dose of 20 mg/kg to induce hepatitis, as previously described [27–29]. All other chemicals and biochemicals used in this study were of high analytical grade.

### 2.2. Experimental Animals

Male Balb/c mice (6–8 weeks old, 20 ± 2 g) were purchased from the Shanghai SLAC Laboratory Animal Co. Ltd (Shanghai, China). The animals were housed in plastic cages with controlled light and dark cycles and fed a standard diet with water in a controlled temperature (25 ± 1°C) and humidity (50 ± 5%) environment. Hepatic injury was elicited in 6–8-week-old male mice by injecting ConA (20 mg/kg body weight or bw) into the tail vein. Resveratrol was given according to dose into three groups (high, medium, and low dose). The mice were randomly divided into six groups of ten mice as follows: (1) saline control group, (2) resveratrol-alone group, (3) ConA-induced model group, (4) low-dose resveratrol + ConA group, (5) medium-dose resveratrol + ConA group, and (6) high-dose resveratrol + ConA group. For the first two weeks, the mice in the resveratrol group received 0.1 mL/10 gbw/day by oral administration. The remaining mice received 0.5% carboxymethyl cellulose solution at 0.1 mL/10 gbw/day. At the fourteenth day, one hour after oral administration, concanavalin-A (0.05 mL/10 g) was injected into the caudal veins of mice, except for the saline and resveratrol-alone groups, which received saline (0.05 mL/10 g). After 8 hours, animals were sacrificed to obtain eye blood. The left hepatic lobes were stored at −80°C until the IL-2, IL-6, and TNF-*α* assays were performed. The right hepatic lobes were fixed in 4% paraformaldehyde at 4°C for hematoxylin-eosin (HE) and immunohistochemical staining. Experiments were performed according to the National Institutes of Health Guidelines for the care and use of laboratory animals and were approved by the Committee on the Ethics of Animal Experimentation of Hirosaki University.

### 2.3. Biochemical Analysis

After blood collection, serum was separated by centrifugation at 3000 rpm for 15 min at room temperature. Serum alanine aminotransferase (ALT) and aspartate aminotransferase (AST) were tested using an automated chemistry analyzer (Olympus AU 1000; Olympus Corporation, Tokyo, Japan).

### 2.4. Western Blot

Protein aliquots were used to determine protein concentration (Bio-Rad Dc Protein Assay; Bio-Rad). Samples were mixed with 4x sample buffer and were electrophoresed on SDS-polyacrylamide gels and transferred electrophoretically onto nitrocellulose paper. Total protein was prepared using standard procedures. The protein concentration in the cleared lysates was determined by the method of Bradford (Bradford, 1976). An equal amount of protein for each treatment was loaded onto 12% polyacrylamide gels; after electrophoresis, the proteins were transferred to nylon membranes. Antibodies were diluted in PBS containing 0.5% BSA. The membranes were later incubated with horseradish peroxidase-conjugated goat anti-rabbit antibody of goat anti-mouse antibody for 1.5 hours at room temperature. After the final wash, membranes were developed with the Odyssey two-color infrared laser imaging system (LI-COR Biosciences, Lincoln, NE, USA) [30].

### 2.5. Histopathologic Examination

Livers from all mice were rinsed with ice-cold saline and a small cross section of the liver was obtained. Liver specimens were fixed in 10% neutral buffered formalin and paraffin embedded, sectioned (4 or 5 *μ*m), and stained with hematoxylin and eosin (H&E). The tissues were examined under a microscope in random order without knowledge of the animal or group.

### 2.6. Immunohistochemistry

Frozen tissue sections were washed with PBS. After being blocked with normal goat serum (Vector Laboratories) for 1 h, sections were incubated for 1 h at room temperature in a dilution of 1 : 100 of Gli-1 and Ptc antibody (Santa Cruz Biotechnology). Sections were washed in PBS, developed with a diaminobenzidine tetrahydrochloride substrate for 15 min, and counterstained with hematoxylin. Slides were then observed by optical microscopy [5, 30]. F4/80 and CD4 in mouse liver tissue were measured by immunohistochemistry (formalin/PFA-fixed paraffin-embedded tissue sections). Sections were fixed in formaldehyde and subjected to heat-mediated antigen retrieval in citrate buffer (pH 6.0) prior to blocking with 1% BSA/PBS and incubated for 1 h 30 min at 25°C.

### 2.7. Statistical Analysis

Statistical analysis was performed using one-way analysis of variance (ANOVA) followed by Tukey-Kramer Test. The values are presented as the means ± the standard error of the mean (SEM) for ten mice in each group. A *p* value of less than 0.05 was considered statistically significant. All statistical analyses were performed using SPSS version 17.0 (SPSS, Inc., Chicago, IL, USA) statistical software.

## 3. Results

### 3.1. Resveratrol Pretreatment Ameliorates ConA-Induced Hepatitis

**Figure 1:**
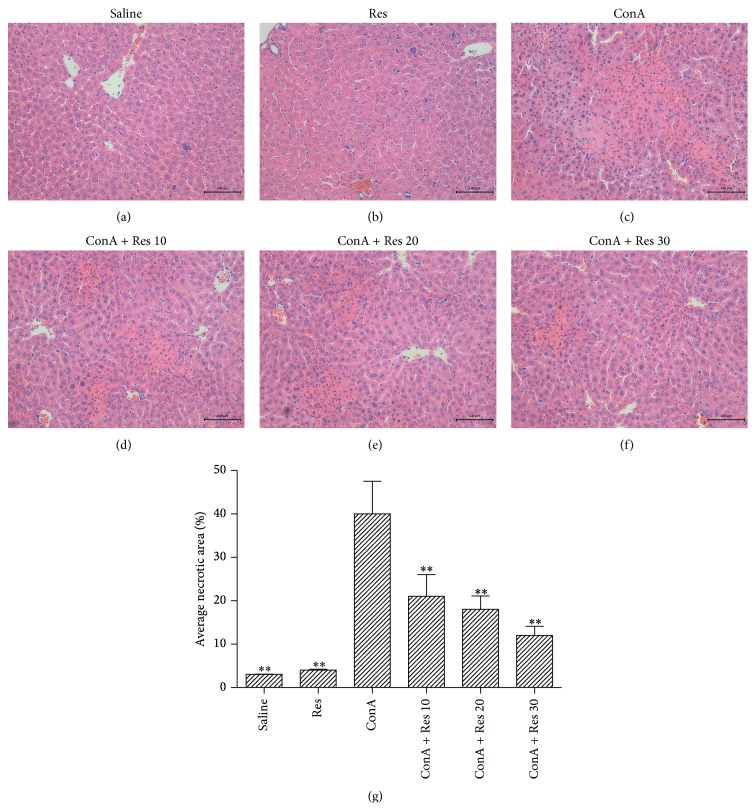
Resveratrol pretreatment attenuates ConA-induced autoimmune hepatitis. Liver histopathology in ConA-induced liver injury. Liver samples were taken from mice treated with ConA (20 mg/kg) and mice treated with ConA and resveratrol (10, 20, and 30 mg/kg, resp., daily oral administration × 14 days). Sections were stained with H&E. (a) Saline group; (b) resveratrol-alone group; (c) ConA group: liver section from a mouse from the ConA-treated group showing extensive liver focal necrosis, portal infiltration, and interface hepatitis. H&E, magnification ×100; ((d)–(f)) ConA + Res (10 mg/kg); ConA + Res (20 mg/kg); ConA + Res (30 mg/kg): liver section from a mouse treated with ConA and resveratrol, and liver histology shows only a minimal grade of cellular infiltration and hepatocyte damage. H&E, magnification ×100. (g) The necrotic areas were analyzed with Image-pro Plus 6.0, indicating statistical significance among groups.

We performed an assay using a ConA-induced hepatitis model and assessed liver function by measuring serum ALT and AST levels. Mice liver tissue of each group was assessed by pathologic examination and Knodell scoring. ALT and AST levels increased after injection of ConA and peak levels decreased in the high-dose resveratrol group. As shown in Table 1, ALT and AST levels clearly increased after ConA injection compared with the saline group at 8 hours (*p* < 0.05); resveratrol pretreatment significantly attenuated the ConA injection-induced elevation of serum ALT and AST (*p* < 0.05). This same result was demonstrated in the histopathologic study and Knodell scores. As shown in Table 1, the Knodell scores of the resveratrol group gradually decreased and there was statistical significance demonstrated between the different concentrations of resveratrol (*p* < 0.01). As shown in Figure 1, we found massive areas of necrosis in the ConA-induced group. In contrast, the resveratrol-treated group showed minor liver damage, indicating that resveratrol pretreatment significantly reduced liver necrosis. Resveratrol administered at 30 mg/kg was more effective. According to the results as analyzed with Image pro Plus 6.0, statistical significance was clearly demonstrated among groups. Thus, resveratrol pretreatment was shown to attenuate ConA-induced autoimmune hepatitis in mice.

### 3.2. Resveratrol Pretreatment Inhibits the Release of Cytokines during ConA-Induced Hepatitis and Expression of Hh Signal Pathway

**Figure 2:**
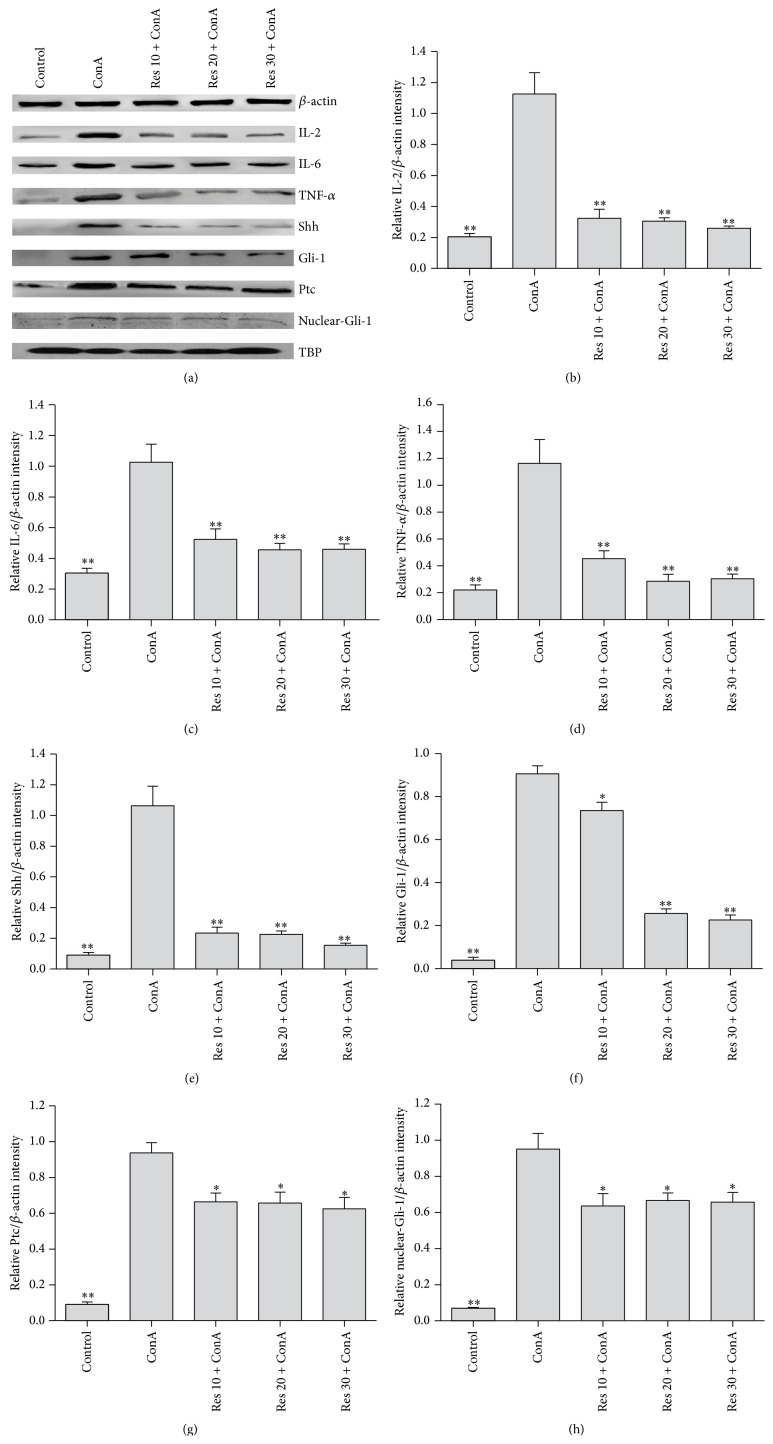
The western blot analysis of IL-2, IL-6, TNF-*α*, Shh, Gli-1, and Ptc after ConA injection in mice and the effects of low (10 mg/kg), medium (20 mg/kg), and high (30 mg/kg) dose resveratrol pretreatment groups at the same time.

ConA-induced hepatitis is associated with changes in the levels of inflammatory cytokines. We demonstrated with immunoblotting that TNF-*α*, IL-2, and IL-6 expression was significantly increased in the ConA model group compared to the saline control group. Resveratrol reduced TNF-*α*, IL-2, and IL-6 production in the ConA-induced mice liver, which is consistent with the degree of liver injury. Next, we examined the effect of resveratrol on Shh, Gli-1, and Ptc expression in the ConA-induced mice liver. Resveratrol pretreatment significantly attenuated Gli-1 and Ptc expression compared to the ConA model group (*p* < 0.05), which was consistent with changes in immunohistochemistry (Figure 2).

### 3.3. Immunohistochemistry Analysis

**Figure 3:**
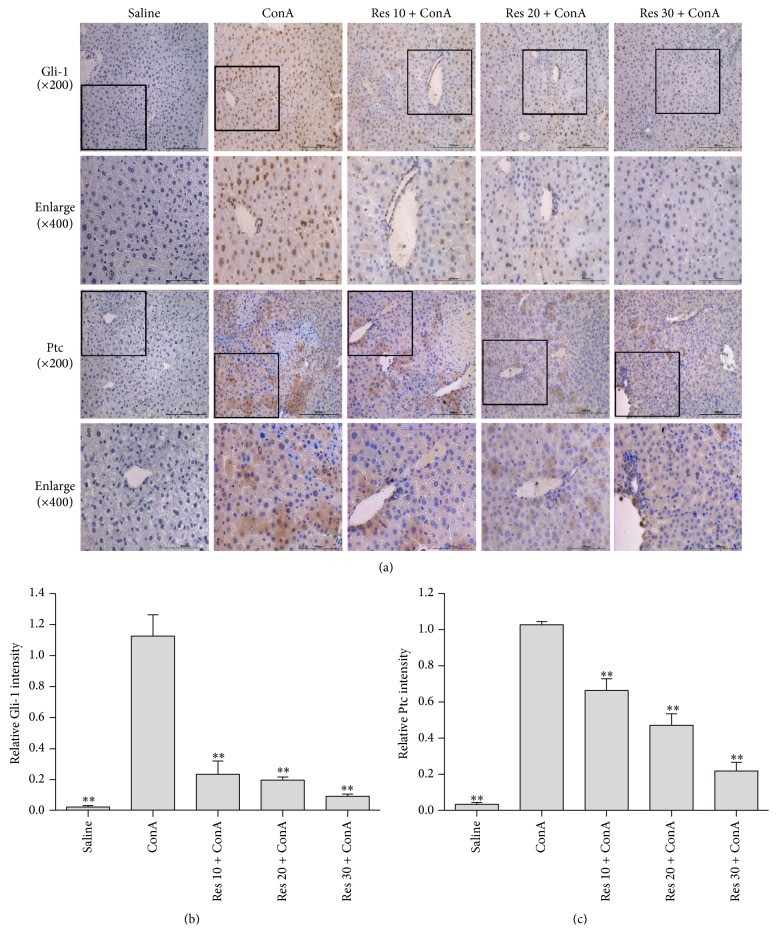
Immunohistochemistry used to detect the expression level of Gli-1 and Ptc 8 hours in all five groups (saline group, ConA model group; ConA + 10 mg/kg Res; ConA + 20 mg/kg Res; ConA + 30 mg/kg Res). Gli-1 and Ptc showed a strong immunoreactivity in ConA sections, but it was weak after Res application in different dose groups.

We hypothesized that the level of Hh pathway activation would increase in parallel with the severity of liver damage. To assess potential correlations between known histologic and clinical predictors of advanced liver disease and Hh pathway activation, immunohistochemistry was performed on liver biopsies. Shh, Gli-1, and Ptc expression, indicators of the activated Hh signal pathway, was detected by immunohistochemistry. It is well known that Gli-1 is more strongly expressed after ConA-induced liver injury. Meanwhile, we found that Gli-1 was mostly located in the nuclei, with some located in the cytoplasm and positively stained brown. Ptc protein was localized in the cytoplasm that showed a brown granular appearance. As shown in Figure 3, Gli-1 was clearly located in the nuclei and was expressed at low levels in the saline group. However, after ConA was induced, Gli-1 was expressed more strongly in the nuclei and had partly translocated to the cytoplasm. In contrast, pretreatment with resveratrol significantly reduced Gli-1 expression in both the nuclei and cytoplasm. In addition, the location and expression of Gli-1 and Ptc by immunohistochemical staining were stronger in the model group than in the saline group at 8 hours (*p* < 0.05). Increased Hh activity (evidenced by accumulation of Hh-ligand producing cells and Hh-responsive target cells) strongly correlated with portal inflammation, ballooning, and fibrosis stage (*p* < 0.0001 for each), supporting a relationship between Hh pathway activation and liver damage (Figure 3).

### 3.4. Resveratrol Reduced the Proliferative Response of T Lymphocytes and Macrophages

**Figure 4:**
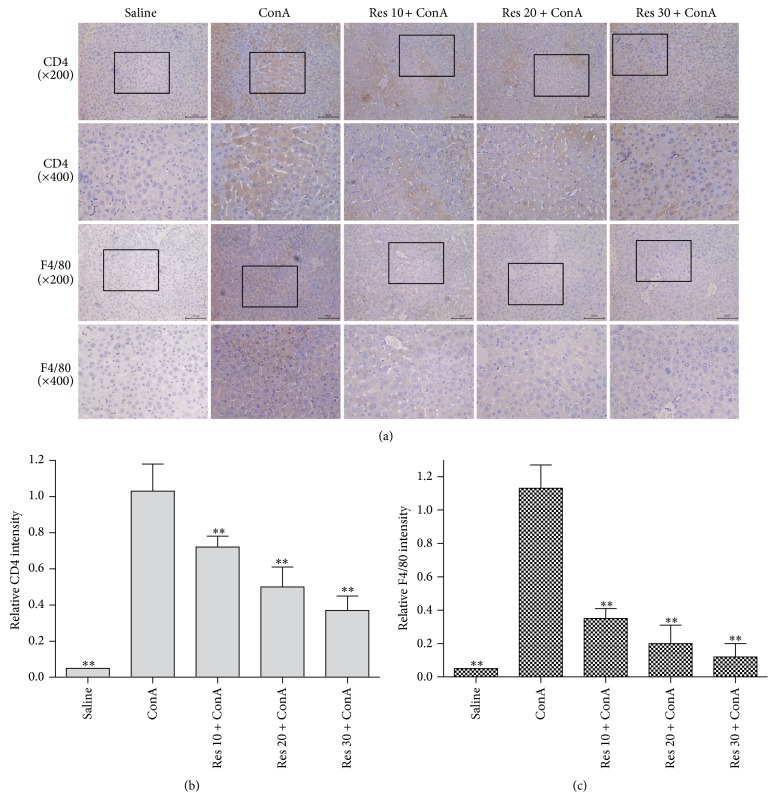
Immunohistochemistry used to detect the expression level of CD4 and F4/80 8 hours in all five groups (saline group, ConA model group; ConA + 10 mg/kg Res; ConA + 20 mg/kg Res; ConA + 30 mg/kg Res). CD4 and F4/80 showed strong immunoreactivity in ConA sections, but it was weak after Res application in different dose groups.

After injection of ConA, serum IL-2, IL-6, and TNF-*α* levels increased dramatically. This suggests that CD4^+^ T helper (Th) cells were involved in liver injury. It was reported that CD4^+^ levels were positive. The cells recognize the ConA-modified major histocompatibility complex (MHC) structures of macrophages and become activated, followed by an inflammation reaction and the release of IL-1 and IL-2 [31]. Cell activation increases the relevant cytokine levels, which leads to liver injury. Meanwhile, we found that resveratrol reduced the proliferative response of T lymphocyte cells. Consistent with this, resveratrol also reduced cytokines such as IL-2. The infiltrate induced by ConA is characterized by macrophages and lymphocytes. In our study, we evaluated the expression of F4/80 as well as T-cell recruitment (i.e., CD4 immunostaining) in different concentrations of resveratrol pretreatment. In the acute ConA model, resveratrol may inhibit macrophages and T helper cells from releasing inflammatory cytokines, which explains why it plays an anti-inflammatory role in the acute model (Figure 4).

## 4. Discussion

Resveratrol has been shown to have a protective effect on liver injury [32]. However, the effects of resveratrol, a powerful and effective antioxidant, on liver injury have not been investigated. Our study is the first to explore the effects of resveratrol on liver injury.

In this study, we used ConA to induce acute liver injury, in order to create a model in which to characterize CD4^+^ T-cell activation, subsequent inflammation, the types of cell death, and liver failure. Our results show that ConA-induced liver injury was ameliorated in resveratrol-pretreated mice and was accompanied by a reduction in the expression of inflammatory cytokines and Hh signaling pathway activation.

Serum ALT and AST levels were markedly elevated in the ConA-treated group compared with saline controls. Serum ALT and AST levels are elevated after hepatocyte death with the release of the transaminases into the blood. Hepatocyte death caused by inflammation was clearly observed with H&E staining (Figure 1). Serum ALT and AST levels were reduced in the resveratrol-pretreated groups relative to those in the ConA-treated groups at 8 hours (Table 1). The areas of cell death in the resveratrol-pretreated group were clearly reduced (Figure 1). Therefore, resveratrol pretreatment clearly attenuated the acute liver injury induced by ConA. The modulatory function of resveratrol in inflammation was reflected in the downregulation of inflammatory cytokines such as IL-2, IL-6, and TNF-*α*. The effect was marked at the protein level, as shown in Figure 2. The anti-inflammatory effects of resveratrol were explicit and consistent with other studies of a variety of inflammatory diseases [5–7]. Both IL-2 and TNF-*α* are produced after macrophage activation and result in an endothelial activation process leading to an increase of adhesion molecule expression, as well as secretion of other cytokines and growth factors. IL-2 and TNF-*α* produce stimulation of acute phase systemic manifestations including somnolence, fever, and changes in liver protein synthesis [33]. Resveratrol decreased the liver laboratory values and the corresponding liver damage—probably as a result of the diminished release of proinflammatory cytokines such as IL-2, -6 and TNF-*α* and a result of T lymphocyte and Kupffer cell activation [33].

The molecular mechanisms by which resveratrol exerts its cytoprotective and anti-inflammatory effects have been attributed to its ROS-scavenging activity, the inhibition of oxygen-free radical formation, and lipid peroxidation, as well as its effects on nitric oxide, modulation of inflammatory cytokines, and chemokines [34]. In addition, resveratrol has been reported to enhance antioxidant enzymes in CCl_4_-induced liver fibrosis and to reduce ethanol-induced oxidative tissue damage in rats [35, 36]. Guy et al. reported that the severity of liver damage parallels the level of Hh pathway activity and the Hh pathway regulates several key aspects of liver repair, including the liver progenitor populations, hepatic recruitment of inflammatory cells, and accumulation of liver myofibroblasts [37].

In the adult organism, Hh signaling remains active and is involved in the regulation of tissue homeostasis, regeneration, and stem cell maintenance [38]. This pathway plays vital roles in tissue morphogenesis during fetal development. It also modulates wound healing responses in a number of adult tissues, including the liver [39–41]. Hh signaling is initiated by a family of ligands (Sonic hedgehog, Shh; Indian hedgehog, Ihh; desert hedgehog, Dhh) which interact with a cell surface receptor (Patched, Ptc) that is expressed on Hh responsive target cells. This interaction depresses the activity of another molecule, Smoothened (Smo), and permits the propagation of intracellular signals that culminate in the nuclear localization of Glioblastoma (Gli) family transcription factors (Gli1, Gli2, and Gli3) that in turn regulate the expression of Gli-target genes [39]. The anti-inflammatory effects of resveratrol are attributed to its binding to members of the Hh pathway that initiate Shh expression and then decrease Gli1 and Ptc expression (Figure 3). Gli-1 and Ptc showed strong immunoreactivity in ConA sections but were weak after resveratrol application in different dose groups.

Immunohistochemistry for F4/80 was used in studies to identify macrophages in mice [42] and since that time has been shown to provide a valid marker of macrophages throughout the body and in a variety of species. In the liver, lymphocytes, sinusoid endothelial cells (SECs), Kupffer cells (KCs), and stellate cells are all involved in the immune response [39]. According to immunohistochemical results, we see that CD4 and F4/80 expression were significantly reduced with different concentrations of resveratrol, a specific inhibitor of the Hh signaling pathway; this indicates that resveratrol reduces inflammation and cell aggregation is likely to inhibit the Hh signaling pathway. It is reported that ConA-induced hepatitis was attenuated by the resveratrol, which inhibited leukocyte infiltration and the expression of T-cell related cytokines and chemokines [43–45]. The result was consistent with our findings (Figure 4).

Several studies have already reported that resveratrol showed anti-inflammatory effects* in vitro* and* in vivo* and inhibits the release of inflammatory cytokines [33, 46]. Nevertheless, the anti-inflammatory effect of resveratrol in ConA-induced liver injury has not yet been reported. In this study, we observed that resveratrol inhibited the expression of inflammatory mediators such as TNF*α*, IL-2, and IL-6. These results indicated that resveratrol was able to improve ConA-induced liver injury by suppressing the inflammatory response in mice.

## 5. Conclusion

We aimed to determine whether resveratrol could inhibit the expression and release of cytokines such as TNF*α*, IL-2, and IL-6 in ConA-induced autoimmune hepatitis. We found the following: (1) resveratrol attenuated ConA-induced autoimmune hepatitis in mice, (2) resveratrol decreases TNF*α*, IL-2, and IL-6 expression* in vivo*, and (3) resveratrol may inhibit the release of Gli-1 and Ptc through modulation of the hedgehog signal pathway. Although there are further mechanisms to be explored, our study provides a new approach for the treatment of acute hepatitis in the clinic.

## Figures and Tables

**Table 1:** Effect of resveratrol on serum biomarkers and Knodell scoring.

Parameter	Group					
	Saline	Res + saline	ConA	Res (10 mg/kg) + ConA	Res (20 mg/kg) + ConA	Res (30 mg/kg) + ConA
ALT (U/L)	30.40 ± 3.45^∗^	32.34 ± 5.34^∗^	389.40 ± 25.60^#^	253.73 ± 14.38^∗#^	239.40 ± 13.83^∗#^	205.52 ± 12.74^∗#^
AST (U/L)	90.60 ± 15.76^∗^	88.60 ± 13.54^∗^	450.74 ± 19.69^#^	250.49 ± 19.34^∗#^	259.89 ± 19.96^∗#^	165.36 ± 21.15^∗#^
Knodell scores	0	0	5.51 ± 1.92^#^	4.67 ± 1.82^∗#^	3.33 ± 1.36^∗#^	3.0 ± 1.52^∗#^

Effects of resveratrol on serum parameters and Knodell scoring in mice with ConA-induced liver injury. Values are expressed as the mean ± standard deviation (*n* = 10). ALT, alanine aminotransferase; AST, aspartate aminotransferase; Res, resveratrol. Statistical significance: ^∗^ *p* < 0.01, ^#^ *p* < 0.01, and ^∗#^ *p* < 0.01 versus saline, respectively. Res alone, ConA alone, ConA + Res (10 mg/kg), ConA + Res (20 mg/kg), and ConA + Res (30 mg/kg). The ConA concentration was 20 mg/kg; resveratrol was administrated by oral gavage.

## Abbreviations

ALT: Alanine aminotransferase
AST: Aspartate aminotransferase
TNF-*α*: Tumor necrosis factor-*α*
IL-2: Interleukin-2
IL-6: Interleukin-6
Shh: Sonic hedgehog
Gli-1: Glioblastoma family transcription factor 1
Ptc: Patched.
